# Underdiagnosis of obstructive lung disease: findings from the French CONSTANCES cohort

**DOI:** 10.1186/s12890-021-01688-z

**Published:** 2021-10-14

**Authors:** Marie-Christine Delmas, Laetitia Bénézet, Céline Ribet, Yuriko Iwatsubo, Marie Zins, Rachel Nadif, Nicolas Roche, Bénédicte Leynaert

**Affiliations:** 1 Santé Publique France, French National Public Health Agency, 12 Rue du Val d’Osne, 94415 Saint-Maurice Cedex, France; 2 Inserm UMS 011, Population-Based Epidemiological Cohorts, Villejuif, France; 3 Inserm, Équipe d’Épidémiologie Respiratoire Intégrative, CESP, Université Paris-Saclay, UVSQ, Université Paris-Sud, Villejuif, France; 4 APHP Centre, Hôpital et Institut Cochin, Service de Pneumologie, Université de Paris, Paris, France

**Keywords:** Obstructive lung disease, Underdiagnosis, COPD, Asthma, France

## Abstract

**Background:**

The burden of undiagnosed obstructive lung disease (OLD) (mainly asthma and chronic obstructive pulmonary disease) is not fully established, and targets for corrective action are yet to be identified. We assessed the underdiagnosis of OLD and its determinants in France.

**Methods:**

CONSTANCES is a French population-based cohort of adults aged 18–69 years at inception. We analysed data collected at inclusion in 2013–2014. Undiagnosed OLD was defined as spirometry-confirmed airflow limitation (FEV_1_/FVC < lower limit of normal) without prior diagnosis of asthma, chronic obstructive pulmonary disease, or bronchiectasis. Multivariate analysis was performed with weighted robust Poisson regression models to estimate the adjusted prevalence ratios (aPR) of undiagnosed OLD.

**Results:**

Spirometry results were available for 19,398 participants. The prevalence of airflow limitation was 4.6%. Overall, 64.4% of adults with airflow limitation did not report a previous diagnosis of OLD. Individuals with high cumulative tobacco consumption (≥ 10 pack-years) (aPR: 1.72 [1.28–2.32]), without respiratory symptoms (aPR: 1.51 [1.28–1.78]), and with preserved lung function (aPR: 1.21 [1.04–1.41] for a 10-point increase in FEV_1_% predicted) had a higher risk of being undiagnosed. Half of symptomatic individuals with airflow limitation (45% of those with moderate to severe airflow limitation) were undiagnosed with OLD.

**Conclusion:**

Underdiagnosis of OLD is very common among French adults, even in patients with respiratory symptoms. Efforts should be made in France to raise awareness about OLD in the general population, improve the detection of respiratory symptoms, and increase the use of spirometry among primary care professionals.

**Supplementary Information:**

The online version contains supplementary material available at 10.1186/s12890-021-01688-z.

## Introduction

Obstructive lung disease (OLD), which mainly relates to asthma and chronic obstructive pulmonary disease (COPD), is both common and serious, representing a growing public health challenge worldwide [1]. Nevertheless, asthma and COPD are frequently undiagnosed. Regarding asthma, population-based studies showed that 20% to 70% of adults with current asthma remained undiagnosed [2]. A pooled analysis of national and international COPD surveys estimated that 81% of COPD patients were undiagnosed [3]. In France, the most recent estimate of the prevalence of current asthma in adults varies from 6 to 9% according to the definition [4]. In the early 2000s, the prevalence of airflow limitation (based on pre-bronchodilator tests) was estimated at 7.5% among non-asthmatic individuals aged 45 years and over [5]. A recent study conducted in middle-aged adults living in two northern French cities showed prevalence estimates ranging from 9.5% to 16.0% according to the city and definition used [6]. More than 70% of those with airflow limitation did not report a diagnosis of OLD.

Undiagnosed OLD is associated with negative health outcomes such as respiratory symptoms, functional impairment and exacerbation, treatment delays, and substantial healthcare utilisation [7–9]. Identifying the factors that contribute to undiagnosed OLD is therefore essential. The present analysis aimed to estimate the prevalence of undiagnosed OLD and identify the risk factors for underdiagnosis in the large French population-based CONSTANCES cohort.

## Methods

### Study population

The methodology of the CONSTANCES cohort study has already been described [10]. For each year of inclusion, individuals aged 18–69 years, affiliated to the main national health insurance covering around 85% of the population, and living in selected administrative areas in France (called “départements”), were randomly chosen to participate in the study according to an unequal probability sampling stratified by gender, age, social category and area. Participants who gave informed consent completed a self-administered questionnaire and underwent a health examination at one of the health prevention centres (HPCs) in the selected areas. Additionally, data were collected for participants and a random sample of non-participants through a linkage to two national databases: the National Health Database (SNDS) that covers all reimbursements for outpatient and hospital healthcare, and the National Retirement Insurance Database that gathers occupational data throughout life. The longitudinal follow-up of participants is still ongoing. This includes annual self-administered questionnaires, health examinations every 4 years, and passive data collection by linkage to the two national databases.

**Fig. 1:**
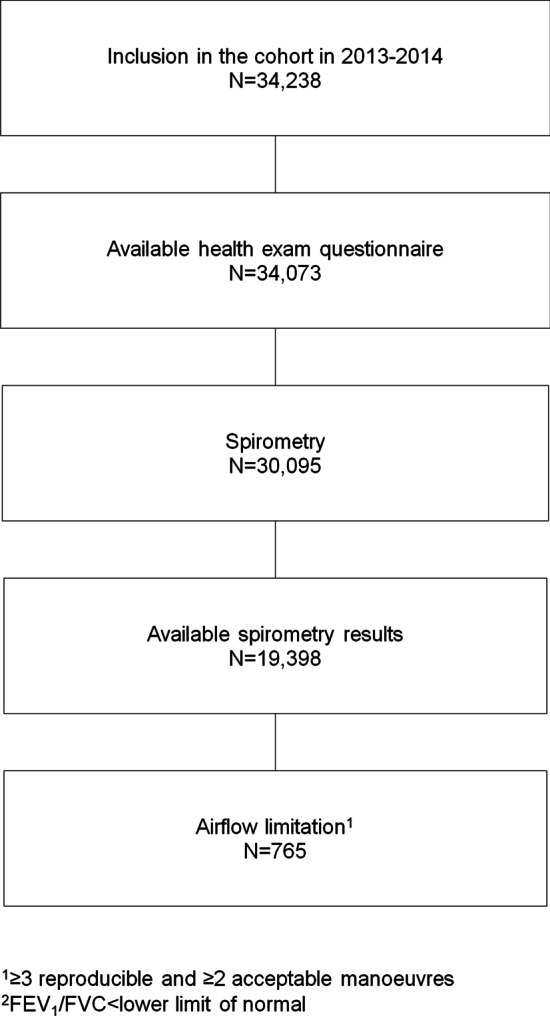
Flow chart of the study population

The present analysis was conducted using data collected at inclusion in 2013–2014. The study population comprised all participants with spirometry results (Fig. 1).

### Data collected at inclusion

The self-administered questionnaire included data on sociodemographic characteristics, occupational history, lifestyle, and health. Questions on respiratory health, taken from the European Community Respiratory Health Survey (ECRHS) questionnaire, covered asthma-like symptoms, chronic bronchitis, and dyspnoea [11]. Dyspnoea was quantified using the modified Medical Research Council scale [12].

Participants were interviewed by a physician about their medical history, including respiratory and cardiovascular diseases, and underwent a comprehensive health examination that included anthropometric measurements, blood pressure, electrocardiogram, vision and hearing tests, spirometry, and blood and urine sampling for biological testing. For participants aged 45 years and older, a specific work-up of functional, physical, and cognitive capacities was performed. To maintain high-quality standards in the measurements, strict quality management was implemented as previously described [13].

### Spirometry and airflow limitation

Spirometry was performed following ATS/ERS guidelines without administering a bronchodilator, as French HPCs are not allowed to administer medication even for diagnostic purposes [14]. For the present study, repeatability criteria were extended to a 200 mL threshold for both forced expiratory volume in 1 s (FEV_1_) and forced vital capacity (FVC). FEV_1_ and FVC were expressed as percentages of predicted values (FEV_1_% predicted and FVC% predicted) using the 2012 Global Lung Initiative (GLI) predictive equations [15]. Airflow limitation was defined using the Global Initiative for Obstructive Lung Disease (GOLD) criteria as FEV_1_/FVC < 0.70 and the lower limit of normal (LLN) criteria with GLI equations. Airflow limitation severity was determined using GOLD criteria (mild: FEV_1_ ≥ 80% predicted; moderate: [50–80%[; severe: [30–50%[; very severe: < 30% predicted) [12].

### Statistical analysis

The outcome was undiagnosed OLD, defined as spirometry-confirmed airflow limitation with no previous diagnosis of OLD (asthma, COPD, emphysema, chronic bronchitis, or bronchiectasis) reported by participants during the medical interview. Participants with undiagnosed OLD were compared to those with diagnosed asthma and COPD for demographic and anthropometric characteristics, education level (coded with the International Standard Classification of Education (ISCED) [16]), lifetime tobacco consumption (none; > 0 and < 10 pack-years; ≥ 10 pack-years), respiratory symptoms (wheezing in the past 12 months, chronic cough or sputum, dyspnoea), cardiovascular history, and spirometry results. Multivariate analysis was performed using robust Poisson regression models to estimate adjusted prevalence ratios of undiagnosed OLD according to the factors studied. Two models were built: the first was adjusted for sociodemographic characteristics (gender, age, education level in two groups of similar size), tobacco consumption, and clinical data (respiratory symptoms, cardiovascular comorbidities), and the second for these features plus FEV_1_% predicted.

All analyses incorporated appropriate weights. Using the sampling weights calculated for each year of inclusion, two consecutive steps of reweighting were performed: the first took into account non-participation in the clinical exam, and the second the unavailability of spirometry results in participants who attended the clinical exam. Reweighting was performed with the equal quantile score method using demographic, socioeconomic, and health-related data, along with inclusion data (tobacco status, presence of respiratory symptoms, body mass index, blood pressure) for the second step [17]. Finally, as 2013 and 2014 samples were independent, annual weights were combined [18]. Statistical analyses were performed using Stata v14 (Stata Corporation, College Station, Texas, USA).

## Results

### Study population and previous diagnoses of OLD

Spirometry results were available (≥ 3 acceptable and ≥ 2 reproducible manoeuvres) for 19,398 out of 34,238 CONSTANCES participants included in 2013 or 2014 (Fig. 1). Overall, 765 participants had FEV_1_/FVC ratio less than LLN, leading to a weighted prevalence of airflow limitation of 4.6% [95% confidence interval: 4.1–5.1%]: 5.2% [4.5–6.0%] in men and 4.0% [3.3–4.6%] in women (*P* = 0.01). Using FEV_1_/FVC ratio < 0.70, the prevalence of airflow limitation was 5.0% [4.5–5.5%]: 6.6% [5.8–7.4%] in men and 3.5% [2.9–4.1%] in women (*P* < 0.001).

In the following analyses, we considered only participants with airflow limitation (FEV_1_/FVC < LLN). Their characteristics are described in Table 1. Women with airflow limitation were younger, were more likely to be never-smokers, and had a better lung function than men. Men with airflow limitation were more likely to report a previous diagnosis of COPD and cardiovascular comorbidities. Overall, a previous diagnosis of OLD (asthma, COPD, emphysema, chronic bronchitis, or bronchiectasis) was reported by 35.6% of individuals with airflow limitation, leading to a proportion of undiagnosed OLD of 64.4%. This proportion did not differ in men and women.

**Table 1:** Characteristics of the study populationparticipants with airflow limitation

	All (N = 765)	Men (N = 385)	Women (N = 380)	*P*
Gender, %				
Men	54.7			
Women	45.3			
Age (years), m	44.4	46.5	41.9	0.002
ISCED education level, %				
0–2 (≤ lower secondary)	14.1	16.9	10.7	0.21
3–4 (upper secondary)	39.5	41.2	37.4	
5–6 (short-cycle tertiary/bachelor)	30.0	27.0	33.5	
7–8 (master/doctorate)	16.5	14.9	18.4	
Tobacco status, %				
Never	31.8	28.9	38.7	0.01
Ever < 10 pack-years	29.5	26.1	33.7	
Ever ≥ 10 pack-years	37.3	45.0	27.5	
Pack-years in smokers, m	18.4	22.9	11.8	< 0.001
BMI (kg/m^2^), m	25.0	25.4	24.5	0.17
Respiratory symptoms, %				
Past-year wheezing	40.3	44.8	34.9	0.08
Chronic cough	17.0	18.8	15.0	0.38
Chronic sputum	12.5	15.1	9.4	0.16
Dyspnoea ≥ grade 2	19.9	16.1	24.6	0.07
≥ 1 symptom	51.9	52.9	50.7	0.69
OLD history, %				
Asthma	29.6	26.0	33.9	0.11
COPD/Emphysema/Chronic bronchitis	8.1	11.4	4.1	0.02
Bronchiectasis	0.1	0.1	0.1	–
≥ 1 OLD	35.6	35.9	35.3	0.91
Cardiovascular history, %				
IHD, stroke, PAD	3.4	5.5	1.0	0.03
Other	11.6	11.8	11.3	
Lung function				
FEV_1_% predicted, m	75.3	72.5	76.4	< 0.001
FVC% predicted, m	92.9	90.4	93.2	0.003
FEV_1_/FVC% predicted	80.5	79.7	80.8	0.003
GOLD severity, %				
Mild	39.2	35.3	44.0	0.01
Moderate	53.5	53.4	53.6	
Severe	7.3	11.2	2.5	

*BMI* body mass index; *COPD* chronic obstructive pulmonary disease; *FEV*_*1*_ forced expiratory volume in 1 s; *FVC* forced vital capacity; *GOLD* Global Initiative on Obstructive Lung Disease; *IHD* ischemic heart disease; *ISCED* International Standard Classification of Education; *OLD* obstructive lung disease; *PAD* peripheral arterial disease; *P P* value for the comparison of men and women

Data are presented as weighted percentage (%) or mean (m)

Missing values in 95 participants (10 for education level, 90 for tobacco status, 5 for BMI, 2 for respiratory symptoms)

### Characteristics of individuals with airflow limitation, with versus without a previous diagnosis of OLD

We investigated the characteristics of individuals who reported a previous diagnosis of asthma or COPD as compared to those with undiagnosed OLD (Table 2). Individuals with undiagnosed OLD had sociodemographic characteristics and smoking habits that ranged between those of individuals with diagnosed asthma and those with diagnosed COPD, although they tended to be closer to those with diagnosed asthma. However, they reported fewer symptoms and had better lung function. Overall, 60.3% of individuals with undiagnosed OLD reported no respiratory symptoms, compared to 27.4% and 20.3% of those with diagnosed asthma and COPD, respectively.

**Table 2:** Comparison of people individuals with diagnosed and undiagnosed obstructive lung disease among thoseparticipants with airflow limitation

	Previous OLD diagnosis^a^			*P*
	Asthma (N = 224)	COPD (N = 46)	No (N = 493)	
Sociodemographic				
Males, %	48.8	77.1	54.5	0.04
Age (years), m	41.8	58.5	43.8	< 0.001
ISCED education level, %				
0–2 (≤ lower secondary)	9.3	37.6	13.4	0.004
3–4 (upper secondary)	36.3	42.1	40.4	
≥ 5 (tertiary)	54.4	20.2	46.2	
Tobacco consumption, %				
Never	43.5	17.5	30.8	< 0.001
Ever < 10 pack-years	37.9	4.7	28.5	
Ever ≥ 10 pack-years	18.6	77.8	40.7	
BMI (kg/m^2^), m	24.6	27.4	24.8	0.38
Respiratory symptoms, %				
Past-year wheezing	64.4	73.8	26.3	< 0.001
Chronic cough	14.8	33.4	16.1	0.10
Chronic sputum	11.8	22.9	11.5	0.29
Dyspnoea grade ≥ 2	20.9	51.8	15.6	< 0.001
≥ 1 symptom	72.6	79.7	39.7	< 0.001
Cardiovascular comorbidities, %				
No	86.7	61.7	87.1	0.003
IHD, stroke, PAD	1.9	6.7	3.7	
Other	11.4	31.8	9.2	
Lung function				
FEV_1_ (L), m	2.56	1.80	2.75	< 0.001
FEV_1_% predicted, m	72.8	58.0	78.5	
GOLD severity, %				< 0.001
Mild	28.3	3.3	48.4	
Moderate	64.9	56.3	48.4	
Severe	6.8	40.4	3.3	< 0.001

*BMI* body mass index; *COPD* chronic obstructive pulmonary disease; *FEV*_*1*_ forced expiratory volume in 1 s; *GOLD* Global Initiative on Obstructive Lung Disease; *IHD* ischemic heart disease; *ISCED* International Standard Classification of Education; *OLD* obstructive lung disease; *PAD* peripheral arterial disease

^a^Previous OLD diagnosis reported by the participants; Asthma: diagnosis of asthma only; COPD: diagnosis of COPD, emphysema, or chronic bronchitis associated with a diagnosis of asthma in eight patients; No: no diagnosis of OLD (asthma, COPD, emphysema, chronic bronchitis, or bronchiectasis)

Data are presented as weighted percentage (%) or weighted mean (m)

Missing values in 95 participants (10 for education level, 90 for tobacco status, 5 for BMI, 2 for respiratory symptoms)

### Symptoms by severity of airflow limitation and previous diagnoses

To investigate whether under-diagnosis might relate to different patterns of symptoms, we explored the relationship between respiratory symptoms and airflow limitation severity, stratified by a previous diagnosis of OLD (Table 3). Among individuals with undiagnosed OLD, the prevalence of respiratory symptoms increased markedly with the severity of airflow limitation (from 28.8% in individuals with mild airflow limitation to 84.1% in those with severe airflow limitation). Among individuals with diagnosed OLD, the proportion of those reporting symptoms was already high (71.2%) in those with mild airflow limitation and increased up to 80.9% in those with severe airflow limitation. Conversely, considering individuals with respiratory symptoms, 49.4% had undiagnosed OLD: 60.8% in those with mild airflow limitation, and 44.9% in those with moderate to severe airflow limitation.

**Table 3:** Respiratory symptoms according to the severity of airflow limitation in participants with and without a previous diagnosis of OLD

	GOLD severity			Total (N = 763)
	Mild N = 319	Moderate N = 4035	Severe N = 41	
No previous OLD diagnosis				
Past-year wheezing, %	19.8	29.4	76.2	26.3
Chronic cough, %	10.1	21.1	28.0	16.1
Chronic sputum, %	6.4	16.4	14.6	11.5
Dyspnoea grade ≥ 2, %	9.1	20.1	44.4	15.6
≥ 1 symptom, %	28.8	47.6	84.1	39.7
Previous OLD diagnosis				
Past-year wheezing, %	58.2	66.3	80.4	66.4
Chronic cough, %	21.3	15.3	30.8	18.9
Chronic sputum, %	13.1	12.2	25.2	14.4
Dyspnoea grade ≥ 2, %	25.2	21.6	57.6	27.8
≥ 1 symptom, %	71.2	73.7	80.9	74.2
Total				
Past-year wheezing, %	27.6	44.6	79.1	40.3
Chronic cough, %	12.4	18.7	29.9	17.0
Chronic sputum, %	7.8	14.7	22.1	12.5
Dyspnoea grade ≥ 2, %	12.4	20.7	53.7	19.9
≥ 1 symptom, %	37.6	58.4	81.8	51.9

*COPD* chronic obstructive pulmonary disease; *GOLD* Global Initiative on Obstructive Lung Disease; *OLD* obstructive lung disease

Data are presented as weighted percentage (%)

### Factors associated with undiagnosed OLD

After adjusting for gender, age, education level, tobacco consumption, respiratory symptoms, and cardiovascular history, only two factors were found to be independently associated with undiagnosed OLD: ever-smokers with a tobacco consumption of ≥ 10 pack-years and individuals without respiratory symptoms had a higher risk of undiagnosed OLD (Table 4, model 1). These associations remained unchanged after further adjustment for FEV_1_% predicted (model 2), and preserved lung function was also independently associated with a higher risk of undiagnosed OLD. Restricting the study population to participants with moderate to severe airflow limitation (FEV_1_ < 80% predicted) or those aged 40 years and over gave similar results (Additional file 1: Tables A.1 and A.2). When restricting the study population to participants under 40 years, however, only the absence of respiratory symptoms was associated with an increased risk of undiagnosed OLD (Additional file 1: Table A.3).

**Table 4:** Factors associated with undiagnosed obstructive lung disease (OLD), multivariate analyses in participants with airflow limitation

	aPR (95% CI)			
	Model 1		Model 2	
Gender				
Men	1		1	
Women	1.12	(0.96–1.30)	1.05	(0.90–1.23)
Age				
For 10-year increase	0.96	(0.89–1.02)	0.98	(0.92–1.05)
ISCED education level				
≤ 4 (≤ upper secondary)	1		1	
≥ 5 (tertiary)	0.92	(0.78–1.08)	0.93	(0.79–1.15)
Tobacco consumption				
Never	1		1	
Ever < 10 pack-years	0.97	(0.79–1.19)	0.95	(0.78–1.14)
Ever ≥ 10 pack-years	1.36	(1.11–1.67)	1.35	(1.11–1.64)
Respiratory symptoms				
≥ 1	1		1	
No	1.67	(1.42–1.97)	1.51	(1.28–1.78)
Cardiovascular comorbidities				
No	1		1	
Yes	0.95	(0.74–1.22)	1.01	(0.79–1.28)
FEV_1_% predicted				
For 10-point increase			1.12	(1.06–1.19)

*aPR* adjusted prevalence ratio; *CI* confidence interval; *FEV*_*1*_ forced expiratory volume in 1 s; *ISCED* International Standard Classification of Education

Weighted robust Poisson regression models adjusted for gender, age, education, tobacco consumption, respiratory symptoms and cardiovascular comorbidities (model 1), plus FEV1 (model 2) in 670 participants with complete data on covariates

## Discussion

This study highlights a high level of OLD underdiagnosis in France. Only 36% of adults with airflow limitation reported a previous diagnosis of OLD. High tobacco consumption, an absence of respiratory symptoms (wheezing, chronic cough or phlegm, dyspnoea), and preserved lung function were associated with a higher risk of being undiagnosed. However, nearly half (45%) of adults with moderate to severe airflow limitation (FEV1/FVC < LLN and FEV_1_ < 80% predicted) remained underdiagnosed with OLD despite experiencing respiratory symptoms.

The major strength of our study is the large sample of the French population, randomly drawn from the general population. We employed reweighting procedures using demographic, socioeconomic, clinical, and healthcare consumption data to correct for non-participation in the study. However, reweighting procedures did not fully correct for the selection bias of the study population. No participants in the CONSTANCES cohort had very severe airflow limitation, which tends to underestimate the prevalence of airflow limitation, and since such severe patients are likely to be diagnosed, to overestimate OLD underdiagnosis.

In our study, airflow limitation was defined based on pre-bronchodilator spirometry, which does not distinguish between asthma and COPD. To estimate the overall prevalence of undiagnosed OLD, pre-bronchodilator (ideally, both pre- and post-bronchodilator) spirometry is more relevant than only post-bronchodilator spirometry, which may miss asthmatic patients with fully reversible airflow limitation. However, lung function testing cannot estimate the rate of asthma underdiagnosis, since many patients, even untreated, can have normal spirometry. Further, pre-bronchodilator spirometry cannot be used to estimate the prevalence and underdiagnosis of COPD, since this diagnosis requires airflow limitation to persist after administering bronchodilators. Another limitation of our study is that diagnosed OLD was defined as a previous diagnosis of asthma, COPD, chronic bronchitis, emphysema, or bronchiectasis reported by participants with airflow limitation, without validating the diagnoses in their medical charts.

We estimated that 64% of French adults with OLD (as identified by pre-bronchodilator airflow limitation) were undiagnosed. Studies assessing OLD underdiagnosis based on pre-bronchodilator spirometry are relatively scarce. In the USA, the analysis of two nationally representative health examination surveys showed that more than 70% of adults aged 20–79 years with obstructive spirometry pattern (pre-bronchodilator FEV_1_/FVC < 0.70) were not diagnosed with asthma or COPD and that underdiagnosis did not change from the first study in 1988–1994 to the second in 2007–2012 [19]. A large underdiagnosis of asthma has been evidenced in both children and adults, including the elderly, with underdiagnosis rates ranging from 20 to 70% [2]. The rate of COPD underdiagnosis appeared to be even higher. Using data from national or international surveys conducted in randomly selected adults aged ≥ 40 years, it was found that 50% to 98% of COPD cases (defined by a post-bronchodilator FEV_1_/FVC < LLN) were undiagnosed, with an average rate of 81% [3]. In France, a study conducted in the early 2000s estimated that 94% of non-asthmatics aged 40 years and over with airflow limitation (pre-bronchodilator FEV_1_/FVC < 0.70) had not been diagnosed with chronic respiratory disease [5]. In northern France in 2011–2013, 72% of middle-aged adults with pre-bronchodilator FEV_1_/FVC < LLN did not report a diagnosis of asthma or COPD [6]. Using the same definition for airflow limitation, we estimated a slightly lower rate (64% overall, and 62% among adults aged 40–69 years).

Several factors may explain the high underdiagnosis of OLD. First, respiratory symptoms may not be present. We found that 48% of individuals with airflow limitation did not report respiratory symptoms (including past-year wheezing, chronic cough or sputum, and dyspnoea grade ≥ 2), with this rate being still high (39%) among those with moderate to severe airflow limitation. However, 49% of individuals with airflow limitation remained underdiagnosed despite the presence of respiratory symptoms. The underreporting of respiratory symptoms to general practitioners by asthmatic patients was shown to contribute significantly to the underdiagnosis of asthma [20]. Regarding COPD, the general population’s limited awareness about this disease and its consequences probably plays an important role in the underreporting of symptoms to general practitioners. In 2017, in France, only 22% of adults reported knowing about COPD, among whom only one-third cited tobacco use as the main cause [21]. Similar figures have been reported worldwide [22–24]. The poor recognition of respiratory symptoms suggestive of COPD by primary care professionals might also play a role. Another reason for the underdiagnosis of OLD is the low use of spirometry. In France, spirometry testing is mainly performed in hospital settings or pulmonologists’ offices. As in many other countries, screening for COPD in the general population is not recommended, but early case finding of COPD is. According to clinical guidelines, spirometry tests should be performed in all patients aged 40 years and over with respiratory symptoms, along with cumulative tobacco exposure of ≥ 15 pack-years or occupational exposure to smoke, gas, or dust [25]. Several initiatives are currently being developed to expand spirometry testing to general practices to decrease the underdiagnosis of COPD, but the results are still pending.

In line with previous population-based studies on COPD underdiagnosis [3, 26], we found that OLD underdiagnosis was more frequent in adults with fewer symptoms and better lung function. COPD is often not recognised in patients with limited respiratory symptoms and preserved lung function, with the diagnosis being delayed until late in the disease process. Studies showed that 20–30% of patients admitted to emergency departments or hospitalised for the first time for COPD exacerbation had not been previously diagnosed or treated [27–29].

We defined airflow limitation using pre-bronchodilator spirometry, which encompasses both persistent and reversible airflow limitation. To increase the specificity of pre-bronchodilator airflow limitation for COPD, we restricted the study population to participants with moderate to severe airflow limitation (FEV_1_ < 80% predicted). It was shown that the proportion of pre-bronchodilator airflow limitation that persists after administering bronchodilators was higher (above 85%) in adults with FEV_1_ < 80% predicted [30]. We also restricted the study population to participants aged 40 years and over, since COPD is extremely rare in young adults. These analyses yielded similar results to those observed for all participants with airflow limitation, with an increased risk of being undiagnosed in those without respiratory symptoms, with preserved lung function, and with a high cumulative tobacco consumption. By contrast, in participants aged under 40 years, no significant association with tobacco consumption was observed. These results could suggest that the association between tobacco consumption and OLD underdiagnosis is related to COPD underdiagnosis.

Despite having less severe airway obstruction and fewer comorbidities than those with a clinical diagnosis of asthma or COPD, people with undiagnosed OLD had a higher mortality risk than the general population [19]. Undiagnosed COPD patients often experience exacerbations, and compared to individuals without COPD, they have impaired health-related quality of life and increased healthcare use [7, 9, 31, 32]. In the absence of diagnosis, these patients cannot be properly managed for their disease, although effective treatments are available to stop smoking, prevent respiratory infections, relieve symptoms, decrease exacerbation risk, and limit quality-of-life impairment [12]. Some treatments were recently shown to decrease mortality in some subpopulations [33, 34]. Regarding asthma, evidence shows that early intervention, particularly with inhaled corticosteroids, has a substantial impact on quality of life and later morbidity [35].

No data on time trends in asthma prevalence among French adults are available, but repeated prevalence surveys among children show an increasing trend [36, 37]. Regarding COPD, increased hospital admissions for COPD exacerbation have been observed [38]. Due to the delay in the mass uptake of smoking in women in the twentieth century, between-gender discrepancies in the current trends for COPD burden are observed. Between 2002 and 2015, the rate of patients hospitalised for COPD exacerbation increased by 100% in women and 30% in men. COPD prevalence modelling in France predicted an increase in prevalence (for both diagnosed and undiagnosed COPD) between 2005 and 2015, especially in women [39]. If the underdiagnosis of OLD is not substantially reduced, the number of undiagnosed individuals will continue to increase in the coming years.

## Conclusion

The underdiagnosis of OLD is very common among adults in France. Many remain undiagnosed despite the significant burden of respiratory symptoms. Underdiagnosis affects not only people with mild airflow limitation but also those with more severe disease. This study highlights the need to reduce the underdiagnosis of OLD in the French population. Efforts should be made to raise awareness about OLD and respiratory symptoms in the general population through national campaigns using various media. Promoting the use of spirometry among primary care professionals along with more systematic screening for symptoms using structured questionnaires should also improve OLD diagnosis.

## Supplementary Information

**Additional file 1.** Underdiagnosis of obstructive lung disease: findings from the French CONSTANCES cohort.

## Data Availability

Access to sensitive and personal data, such as those of the CONSTANCES cohort, is restricted by French law. The CONSTANCES coordination team makes the data available, upon request, to qualified researchers who have obtained prior authorization from the French national data protection authority (Commission nationale de l’informatique et des libertés, CNIL). Information for applicants to CONSTANCES data is available on the website: https://www.constances.fr/CFP.pdf. CONSTANCES investigators may be contacted at the following address: contact@constances.fr.

## Abbreviations

ATS: American Thoracic Society
COPD: Chronic obstructive pulmonary disease
ERS: European Respiratory Society
HPC: Health prevention centres
FEV_1_: Forced expiratory volume in 1 s
FVC: Forced vital capacity
GLI: Global Lung Function Initiative
GOLD: Global Initiative on Obstructive Lung Disease
OLD: Obstructive lung disease
