# Supplementary material for: Underdiagnosis of obstructive lung disease: findings from the French CONSTANCES cohort
Source: BMC Pulm Med. 2021 Oct 14;21:319. doi: 10.1186/s12890-021-01688-z (PMC8518215; doi:10.1186/s12890-021-01688-z)
Supplement: Supplementary file 1 — Additional file 1. Underdiagnosis of obstructive lung disease: findings from the French CONSTANCES cohort. [file 12890_2021_1688_MOESM1_ESM.docx]

**Table A.1: Factors associated with undiagnosed obstructive lung disease (OLD), multivariate analyses in participants with moderate to severe airflow limitation**

|  | **aPR (95% CI)** | | | |
| --- | --- | --- | --- | --- |
|  | **Model 1** | | **Model 2** | |
| Gender  Men  Women | 1  1.14 | (0.89–1.45) | 1  1.06 | (0.83–1.35) |
| Age  For 10-year increase | 0.95 | (0.85–1.05) | 0.98 | (0.88–1.09) |
| ISCED education level  ≤4 (≤upper secondary)  ≥5 (tertiary) | 1  0.97 | (0.76–1.25) | 1  0.97 | (0.75–1.25) |
| Tobacco consumption  Never  Ever <10 pack-years  Ever ≥10 pack-years | 1  0.90  **1.75** | (0.60–1.34)  **(1.27–2.40)** | 1  0.87  **1.72** | (0.58–1.32)  **(1.28–2.32)** |
| Respiratory symptoms  ≥1  No | **1**  **1.86** | **(1.49–2.33)** | **1**  **1.73** | **(1.38–2.17)** |
| Cardiovascular comorbidities  No  Yes | 1  1.04 | (0.77–1.41) | 1  1.06 | (0.79–1.42) |
| FEV_1_% predicted  For 10-point increase |  |  | **1.21** | **(1.04–1.41)** |

Abbreviations: aPR = adjusted prevalence ratio; CI = confidence interval; FEV_1_ = forced expiratory volume in 1 second; ISCED = International Standard Classification of Education

Weighted robust Poisson regression models adjusted for gender, age, education, tobacco consumption, respiratory symptoms and cardiovascular comorbidities (model 1), plus FEV1 (model 2) in 382 participants with moderate to severe airflow limitation and complete data on covariates.

**Table A.2: Factors associated with undiagnosed obstructive lung disease (OLD), multivariate analyses in participants aged 40-69 years**

|  | **aPR (95% CI)** | | | |
| --- | --- | --- | --- | --- |
|  | **Model 1** | | **Model 2** | |
| Gender  Men  Women | 1  1.10 | (0.90–1.35) | 1  1.01 | (0.82–1.23) |
| Age  For 10-year increase | 1.01 | (0.91–1.13) | 1.04 | (0.93–1.17) |
| ISCED education level  ≤4 (≤upper secondary)  ≥5 (tertiary) | 1  0.86 | (0.69–1.08) | 1  0.94 | (0.76–1.16) |
| Tobacco consumption  Never  Ever <10 pack-years  Ever ≥10 pack-years | 1  1.27  **1.62** | (0.93–1.73)  **(1.21–2.16)** | 1  1.16  **1.60** | (0.88–1.54)  **(1.22–2.09)** |
| Respiratory symptoms  ≥1  No | **1**  **1.60** | **(1.32–1.94)** | **1**  **1.41** | **(1.17–1.69)** |
| Cardiovascular comorbidities  No  Yes | 1  0.92 | (0.70–1.20) | 1  0.97 | (0.75–1.27) |
| FEV_1_% predicted  For 10-point increase |  |  | **1.15** | **(1.07–1.23)** |

Abbreviations: aPR = adjusted prevalence ratio; CI = confidence interval; FEV_1_ = forced expiratory volume in 1 second; ISCED = International Standard Classification of Education

Weighted robust Poisson regression models adjusted for gender, age, education, tobacco consumption, respiratory symptoms and cardiovascular comorbidities (model 1), plus FEV1 (model 2) in 427 participants aged 40-69 years and complete data on covariates.

**Table A.3 Factors associated with undiagnosed obstructive lung disease (OLD), multivariate analyses in participants aged 18-39 years**

|  | **aPR (95% CI)** | | | |
| --- | --- | --- | --- | --- |
|  | **Model 1** | | **Model 2** | |
| Gender  Men  Women | 1  1.15 | (0.91–1.45) | 1  1.13 | (0.89–1.43) |
| Age  For 10-year increase | 1.15 | (0.91–1.47) | 1.13 | (0.89–1.43) |
| ISCED education level  ≤4 (≤upper secondary)  ≥5 (tertiary) | 1  0.93 | (0.73–1.18) | 1  0.92 | (0.72–1.17) |
| Tobacco consumption  Never  Ever <10 pack-years  Ever ≥10 pack-years | 1  0.80  1.02 | (0.64–1.01)  (0.72–1.44) | 1  0.80  1.01 | (0.64–1.01)  (0.71–1.44) |
| Respiratory symptoms  ≥1  No | **1**  **1.67** | **(1.29–2.17)** | **1**  **1.61** | **(1.22–2.13)** |
| Cardiovascular comorbidities  No  Yes | 1  0.98 | (0.70–1.38) | 1  1.01 | (0.71–1.45) |
| FEV_1_% predicted  For 10-point increase |  |  | 1.05 | (0.96–1.15) |

Abbreviations: aPR = adjusted prevalence ratio; CI = confidence interval; FEV_1_ = forced expiratory volume in 1 second; ISCED = International Standard Classification of Education

Weighted robust Poisson regression models adjusted for gender, age, education, tobacco consumption, respiratory symptoms and cardiovascular comorbidities (model 1), plus FEV1 (model 2) in 243 participants aged 18-39 years and complete data on covariates.
